# Point Prevalence Survey of Antimicrobial Use and Resistance during the COVID-19 Era among Hospitals in Saudi Arabia and the Implications

**DOI:** 10.3390/antibiotics12111609

**Published:** 2023-11-09

**Authors:** Abdul Haseeb, Safa S. Almarzoky Abuhussain, Saleh Alghamdi, Shahad M. Bahshwan, Ahmad J. Mahrous, Yazeed A. Alzahrani, Albaraa Faraj Alzahrani, Abdullmoin AlQarni, Manal AlGethamy, Asem Saleh Naji, Asim Abdulaziz Omar Khogeer, Muhammad Shahid Iqbal, Brian Godman, Zikria Saleem

**Affiliations:** 1 Department of Pharmacy Practice, College of Pharmacy, Umm Al-Qura University, Makkah 24382, Saudi Arabia; 2 Department of Clinical Pharmacy, Faculty of Pharmacy, Al-Baha University, Al-Baha 57911, Saudi Arabia; 3 Pharmacy Department, Armed Forces Hospitals Southern Region, Khamis Mushayte 62411, Saudi Arabia; 4 Infectious Diseases Department, Alnoor Specialist Hospital, Makkah 24382, Saudi Arabia; 5 Department of Infection Prevention and Control Program, Alnoor Specialist Hospital, Makkah 24382, Saudi Arabia; 6 Plan and Research Department, Ministry of Health (MOH), Makkah 12211, Saudi Arabia; 7 Medical Genetics Unit, Maternity & Children Hospital, Makkah Healthcare Cluster, Ministry of Health, Makkah 24382, Saudi Arabia; 8 Department of Clinical Pharmacy, College of Pharmacy, Prince Sattam Bin Abdulaziz University, Alkharj 16242, Saudi Arabia; 9 Department of Public Health Pharmacy and Management, School of Pharmacy, Sefako Makgatho Health Sciences University, Pretoria 0208, South Africa; 10 Department of Pharmacoepidemiology, Strathclyde Institute of Pharmacy and Biomedical Science (SIPBS), University of Strathclyde, Glasgow G4 0RE, UK; 11 Department of Pharmacy Practice, Faculty of Pharmacy, Bahauddin Zakariya University, Multan 60800, Pakistan

**Keywords:** antibiotics, antimicrobial resistance, AWaRe classification, hospitals, point prevalence survey, Saudi Arabia

## Abstract

The inappropriate prescribing of antimicrobials increases antimicrobial resistance (AMR), which poses an appreciable threat to public health, increasing morbidity and mortality. Inappropriate antimicrobial prescribing includes their prescribing in patients hospitalized with COVID-19, despite limited evidence of bacterial infections or coinfections. Knowledge of current antimicrobial utilization in Saudi Arabia is currently limited. Consequently, the objective of this study was to document current antimicrobial prescribing patterns among Saudi hospitals during the COVID-19 pandemic. This study included patients with or without COVID-19 who were admitted to five hospitals in Makkah, Saudi Arabia. Data were gathered using the Global PPS methodology and analyzed using descriptive statistics. Out of 897 hospitalized patients, 518 were treated with antibiotics (57.7%), with an average of 1.9 antibiotics per patient. There were 174 culture reports collected, representing 36.5% of all cases. The most common indication for antibiotics use was community-acquired infections, accounting for 61.4% of all cases. ‘Watch’ antibiotics were the most commonly prescribed antibiotics, with the cephalosporins and carbapenems representing 38.7% of all antibiotics prescribed, followed by the penicillins (23.2%). Notably, Piperacillin/Tazobactam and Azithromycin were prescribed at relatively higher rates for COVID-19 patients. These findings highlight the need for continuous efforts to optimize the rational use of antibiotics through instigating appropriate antimicrobial stewardship programs in hospitals and, as a result, reduce AMR in the country.

## 1. Introduction

The prescribing and dispensing of antimicrobials in situations where they are not required, when alternative treatments are more appropriate, or when a broad-spectrum antibiotic is being prescribed when a narrow one is adequate, have enhanced antimicrobial resistance (AMR) [1,2,3,4]. When bacteria become resistant to antibiotics, they can continue to grow and multiply, leading to infections that are more difficult to treat [5]. This needs to be avoided, as AMR increases the number and severity of infections, as well as increasing morbidity, mortality, and healthcare costs [3,6,7,8]. As a result of its growing impact on morbidity and mortality, AMR is increasingly being considered as the next pandemic after COVID-19 [9,10].

The coronavirus disease (COVID-19) was first identified in December 2019 in Wuhan, China, when a group of patients with pneumonia of unknown causes were found to be infected with a new type of coronavirus called SARS-CoV-2 [11,12,13]. The COVID-19 outbreak, caused by SARS-CoV-2 infection, rapidly spread beyond Wuhan, and has since become a worldwide pandemic [14]. The first case of COVID-19 in Saudi Arabia was reported on 2 March 2020 [15]. The spread of COVID-19 in Saudi Arabia has been influenced by government public health measures and population compliance, as well as the emergence of new variants more recently [16,17,18]. The initial measures introduced by the government in Saudi Arabia helped to slow the spread of the virus, similar to other countries [17,19]. However, cases have increased over time, with some periods of rapid transmission and others where the rate of transmission has been slower. New variants have also contributed to the spread of the virus [20].

A number of studies and systematic reviews have documented extensive prescribing of antibiotics in patients hospitalized with COVID-19, despite limited evidence of bacterial coinfections or secondary infections [21,22,23,24]. There is a concern that high rates of prescribing of antibiotics in patients hospitalized with COVID-19 might spill over to their prescribing in patients hospitalized during the recent pandemic for other reasons apart from COVID-19 [21]. Consequently, further research is needed to understand the patterns and reasons for antimicrobial use in patients admitted to hospital for a variety of reasons during the COVID-19 pandemic. There is a need to develop strategies to optimize antimicrobial prescribing if issues and concerns are identified to reduce the threat of increasing AMR [25,26].

A recent review documenting patterns of antibiotic prescribing in hospitals among Middle Eastern countries documented five published studies from Saudi Arabia using the point prevalence survey (PPS) methodology [27]. Point prevalence surveys (PPSs) are a tool used to collect data on the prevalence and characteristics of a specific condition or exposure to antimicrobials typically in hospitals at a particular point in time [28,29,30,31,32,33,34]. Consequently, PPS studies do not typically include seasonal variation; however, they can be repeated to ascertain whether there have been any changes in prescribing patterns over time, including changes in the rate of empiric prescribing; alternatively, changes in conversion from IV to oral therapy where pertinent [32,33,34,35,36]. However, PPS studies can be broken down into specific populations including adults and children, as well as among specific groups, including patients admitted to emergency departments in Saudi Arabia [34,36,37,38,39,40]. In the case of children, this may include prevalence rates broken down by specific age groups; generally though, this is not the case as seen in the global PPS study and studies among children in Pakistan, as well as the management of children with COVID-19 admitted to hospitals in Bangladesh and India [36,38,39,41,42]. PPS studies are also not time-series analyses, which typically investigate specific policies or activities over time, including policies to limit the utilization of antibiotics, or alternatively, the impact of events such as COVID-19 on the utilization of antimicrobials in ambulatory care [43,44,45].

The PPS studies conducted to date among Saudi hospitals have shown appreciable variation in the prescribing of antimicrobials. Antibiotic prescribing ranged from as low as 14.4% of hospitalized patients up to 61.9% of hospitalized patients, depending on the setting and the characteristics of the patients [29,30]. Documented prevalence rates in the other studies ranged from 40% to 49.2% [31,32,33]. An appreciable worry is that universally, there was considerable prescribing of ‘Watch’ and ‘Reserve’ antibiotics in these studies [29,30,31,32,33]. This is a concern, since there should be limited prescribing of antibiotics in the “Watch” group, as these antibiotics are considered to have greater resistance potential and toxicity [46,47,48,49]. In addition, there should be very limited prescribing of antibiotics in the “Reserve” antibiotics, since these are considered antibiotics of last resort [46,47,48,49]. Overall, the target should be at least 60% of antibiotics prescribed in hospitals should be from the “Access” list [49]. High AMR rates are already a concern in Saudi Arabia, as seen in published studies documenting high rates of extended-spectrum (ES) ß-lactamase *Escherichia coli* (*E. coli*), ES cephalosporin-resistant *Klebsiella* spp., and MRSA [30,50,51,52]. Consequently, antimicrobial prescribing rates in Saudi Arabian hospitals need to be urgently addressed, including during the pandemic, to reverse growing rates of AMR in the Kingdom. Antimicrobial stewardship programs (ASPs) are seen as effective initiatives to reduce AMR, as they seek to optimize subsequent prescribing of antimicrobials, including antibiotics, in hospitals to reduce the risk of AMR, while improving patient outcomes [53,54,55]. There have, though, been concerns with the challenges associated with the implementation of ASPs across countries [56]; however, this is now changing, as seen by an appreciable number of ASPs successfully introduced across the Middle East in recent years, including Saudi Arabia [27,57,58]. The key to successfully implementing ASPs, including among hospitals in Saudi Arabia, includes top management support alongside the necessary education and training [58,59]. However, there can be difficulties if different guidelines are currently circulating [60]. The introduction of the AWaRe book covering suggested management for a range of infections should help to address such concerns [46,47].

Conducting PPS studies is in line with the goals of the World Health Organization (WHO), which has emphasized the importance of careful and responsible use of antimicrobials, including antibiotics, in the management of patients with infectious diseases. This includes those with COVID-19, as antimicrobials are not expected to have an impact on the viral infection itself, and may well contribute to the development of AMR [61]. However, to the best of our knowledge, no PPS has been undertaken among Saudi hospitals to assess the state of antimicrobials use in hospitalized patients in the COVID-19 era. This includes the prevalence and characteristics of antimicrobial use, as well as the indications for their prescribing, including their appropriateness. Consequently, this study was undertaken to address this information gap. The findings are likely to provide valuable information on current prescribing patterns to inform efforts, including the instigation of pertinent ASPs, to optimize future antimicrobial use in the country and reduce the risk of AMR.

## 2. Results

Out of 897 hospitalized patients, the number of patients treated with antibiotics was 518 (57.7%). The total number of antibiotics prescribed was 982, with an average of 1.9 antibiotics prescribed per patient. Just over half of the patients (54.1%) were prescribed single antibiotic therapy. A total of 50.2% (260) of antibiotics were prescribed for male patients. The average age of surveyed patients was 34.3 years with a median of 33 days, and average length of hospitalization was 10.8 days with a median of 7 days (Table 1). The number of patients with comorbidities (31.7%), currently on immunosuppressant (5.4%), undergoing surgical procedures (30.1%) as well as the extent of catheterization are also documented in Table 1.

Table 2 presents data on culture reports, samples taken, and resistance microbes found among the hospitalized patients. A total of 174 culture reports were obtained, which represented 36.5% of all cases. Samples were taken from various sources including blood (116, 22.4%), cerebrospinal fluid (CSF) (6, 1.2%), sputum (8, 1.5%), urine (24, 4.6%), and wound swabs (20, 3.9%). Various resistance pathogens were found among the hospitalized patients. These included carbapenem-resistant nonfermenter Gram-negative (20, 3.9%), ESBL-producing nonfermenter Gram-negative bacilli (8, 1.6%), and third-generation cephalosporin-resistant pathogens (22, 4.2%). Overall, a diverse range of resistant pathogens was identified among the participating patients in the various hospitals in Saudi Arabia, with varying frequencies of occurrence. The most common indication for prescribing antimicrobials among the studied population was community-acquired infection, which accounted for 61.4% of all cases. Hospital-acquired infections also represented a significant portion of indications (30.5% of all cases). Other indications for antimicrobial use among participating hospitals included surgical (4.2%) and medical prophylaxis (3.9%). The most common organ systems for which antibiotics were prescribed included blood (23.2%), respiratory (22%), and skin, soft tissue, bone, and joint infections (16.6%). Overall, there was a diverse range of indications for the use of antimicrobials among participating hospitals with varying frequencies of occurrence.

A number of different classes of antibiotics were prescribed among the hospitalized patients. The most commonly prescribed class was the cephalosporins and carbapenems (J01D) (38.7% of all cases). This was followed by the penicillins (J01C) at 23.2% of all cases, and the aminoglycosides (J01G) (6.5%). This indicates that a diverse range of antibiotics was prescribed, with varying frequencies in the different classes. Table 3, Table 4, Table 5 and Table 6 document the extent of individual antibiotics prescribed, including their AWaRe classification. The most commonly prescribed antibiotics were all ‘Watch’ antibiotics, e.g., ceftriaxone (13.9% of all cases), piperacillin/tazobactam (12.5%), vancomycin (10.8%), and meropenem (8.4% of all cases). The first ‘Access’ antibiotic was cefazolin, representing 7.6% of all cases. Colistin, a ‘Reserve’ antibiotic, was also prescribed, but its frequency was low, at 1.4% of cases. The category of “Miscellaneous” antibiotics within our study encompasses a variety of antimicrobial agents, each with its own distinctive characteristics. Among these, trimethoprim/sulfamethoxazole (A), Clarithromycin (W), and Imipenem (W), were notable, with quantities of 10, 8, and 8, respectively. Furthermore, Doxycycline (A), Ertapenem (W), Moxifloxacin (W) Linezolid (R), and Tigecycline (R) each appeared in our dataset, with quantities ranging from 2 to 8.

The data presented in the Table also shed light on the gender-wise utilization of antibiotics, offering insights into prescription patterns among male and female patients. Ceftriaxone, vancomycin, and ampicillin are more frequently prescribed to female patients, with 14.81%, 11.11%, and 10.70% of total prescriptions, respectively, compared to their male counterparts. These gender-based disparities in antibiotic prescription rates could be influenced by a number of factors, including differences in the types of infections prevalent among male and female patients, physiological dissimilarities, and prescribing practices among healthcare providers. Moreover, the tables provide an overview of the most commonly prescribed antimicrobial drugs for COVID-19 cases. Notably, Piperacillin/Tazobactam and Azithromycin were prescribed at relatively higher rates for COVID-19 patients.

In the context of antibiotic usage, several notable trends emerge when comparing community-acquired infections to hospital-acquired infections. Ceftriaxone (W) and Piperacillin/Tazobactam (W) are among the most commonly prescribed antibiotics in both settings, with percentages of 16.4% and 13.1%, respectively, for community-acquired infections, and 8.5% and 12.1%, respectively, for hospital-acquired infections. Interestingly, Vancomycin (W) and Meropenem (W) exhibit higher utilization rates in hospital-acquired infections (17.11% and 13.3%, respectively) compared to community-acquired infections (8.1% and 6.4%, respectively). These data provide insights into the differing patterns of antibiotic usage in these two clinical contexts. Notable findings include Ceftriaxone (W) being prominently used in respiratory infections, while Piperacillin/Tazobactam (W), Vancomycin (W), and Meropenem (W) demonstrate significant utilization in bloodstream infections.

## 3. Discussion

In this study, we document the use of antibiotics among several public sector hospitals in Saudi Arabia during the pandemic. This including patients with suspected or confirmed COVID-19 infections, as well as those admitted without COVID-19 infections for a variety of other indications. The point prevalence of antibiotic prescribing was 57.7%. This is typically higher that the rates reported in previous studies conducted in Saudi Arabia where, as mentioned, prevalence rates ranged from 14.4% of patients up to 61.9% of patients [29,30]. However, because this study was undertaken during the current COVID-19 epidemic, comparing the data amongst the numerous investigations is challenging. In spite of this, antibiotic prescribing rates were comparable to recent PPS research conducted in Pakistan during the pandemic, which found that 64.6% of hospitalized patients were prescribed antibiotics [21]. In addition, each patient was prescribed on average 1.9 antibiotics, which is similar to the rate of 1.76 antibiotics per patient in Pakistan and others [21,30,34]. Alongside this, among low- and middle-income countries (LMICs) there are generally higher prescribing rates of antimicrobials [49,62], which is a concern.

In our investigation, culture reports were obtained for 36.5% of patients, with blood being the most prevalent sample source (22.4%). Encouragingly, this is higher than some previous pre-pandemic studies conducted in Saudi Arabia where only 18.6% of culture tests were performed [40]. In addition, this is appreciably higher than seen in a number of African countries, especially if patients are required to help fund sensitivity tests [62]. This may represent increasing expectations of testing patients as a result of the pandemic. However, we cannot say this with certainty, and more information is needed to understand the reasons for this increase in order to further improve testing rates. Hopefully, this will continue in Saudi Arabia aiding appropriate empiric prescribing. This contrasts with countries where there is currently limited surveillance of resistance patterns [62].

A concern in our study is that third-generation cephalosporins were the most resistant category of antibiotics. These findings are perhaps not surprising, though, given the appreciable prescribing of cephalosporins among the studied hospitals, and correlate with studies depicting the prevalence of extended-spectrum β-lactamases (ESBLs), which adversely affect the potency of cephalosporins in Saudi Arabia [50].

Of equal concern was the high rate of prescribing of ‘Watch’ antibiotics in our study, with the four most prescribed antibiotics all in the ‘Watch’ group, which represented 45.6% of the antibiotics prescribed. Overall, the cephalosporins and carbapenems were the most prescribed class of antibiotics, representing 38.70% of all cases, followed by penicillins, representing 23.22% of all cases. While these findings correlate with previous documented findings regarding the prescribing of antimicrobials during COVID-19 in Egypt and Pakistan with minor variations [21,63], the appreciable prescribing of ‘Watch’ antibiotics is a concern because of its impact on AMR [2,49]. This calls for the urgent introduction of pertinent ASPs in target hospitals to improve future prescribing [27,54,62,64]. The target for ‘Access’ antibiotics should be 60% of all antibiotics prescribed, building on the recent publication of the ‘AWaRe’ book, with suggested antibiotics for a range of indications [2,46,47].

Encouragingly, a number of ASPs have already been undertaken in Saudi Arabia, acting as exemplars to provide future guidance [57,65,66,67], combined with examples from a number of other hospitals in the Middle East. Assessing antibiotic usage is a crucial aspect of ASP initiatives to enhance future appropriate use of antibiotics. We will be working with target hospitals in Saudi Arabia in the future to identify additional ways of improving their appropriate antibiotic use, and following these through with ASPs. This is the only way to reduce AMR in these hospitals in line with the goals of the National Action Plan to reduce AMR [68]. The recently available AWaRe book will be helpful when directing and monitoring the appropriateness of prescribed antibiotics, especially given previous concerns [46,47,60], in addition to producing accurate antibiograms among participating hospitals based on current sensitivity patterns. This is essential when seeking to improve empiric prescribing, and calls for an increase in sensitivity testing among hospitals in Saudi Arabia in the future, building on encouraging signs.

We are aware of a number of limitations of our study. Firstly, we did not assess the appropriateness of antibiotic prescribing use among all patients, including those with COVID-19, in Saudi Arabia. Similarly, our study relied solely on the information documented in the patients’ notes, without checking any interaction between the prescribed antibiotics. However, this is normal in PPS studies. We also did not stratify patients by age category, which is similar to the results reported in global PPS studies [37,38]. In addition, we did break the patients down into clinic type, preferring to concentrate on the clinical indications for the reasons stated. We also concentrated on public hospitals for the reasons stated. As a result, the hospitals that participated in the study may not fully represent the current situation in Saudi Arabia, since a number of approached hospitals were unable to participate due to various reasons. Moreover, the PPS study has another limitation, in that it is a cross-sectional study, meaning that all measurements were made at only one time point. This means that seasonal effects, trends, and interactions among factors that could influence antimicrobial treatment and resistance cannot be analyzed. Time-series analyses could not be performed. Nonetheless, we believe that this study provides valuable insights into the patterns of antibiotic use among hospitals in Saudi Arabia during the COVID-19 pandemic, and identifies key areas for future ASPs. We intend to follow up on these findings in our future research endeavors.

## 4. Materials and Methods

### 4.1. Study Design and Settings

Using the Global PPS approach, a PPS of antibiotic use was carried out in Makkah region hospitals under the Ministry of Health [37]. This study covered both COVID-19 and non-COVID-19 patients admitted to participating hospitals between 28 March 2022, and 20 September 2022. We did not differentiate between these two patient groups, as we were primarily interested in overall antibiotic prescribing rates now that the pandemic is more manageable, especially with the availability of effective vaccines [69,70,71]. However, the overuse of antibiotics in patients admitted to hospital with COVID-19 seen worldwide in the early years of the pandemic may still persist [22,23,24].

A number of hospitals in Makkah City in Saudi Arabia were invited to participate in this PPS study through a purposeful sampling approach, to reflect the healthcare system in Saudi Arabia. This included both public and private hospitals in Saudi Arabia, similar to studies in other countries with mixed healthcare systems. Makkah city was conveniently chosen for this initial study in view of access to hospitals, as difficulties were encountered with accessing patients in a number of other hospitals during the pandemic.

Eventually, five public hospitals were chosen for this initial study, as the public system accounts for the majority of the population in Saudi Arabia, with public hospitals currently offering greater accessibility to specialized care, often at no cost or at affordable costs to patients compared with private hospitals. Consequently, public hospitals are more likely to have greater variability in their populations, providing greater guidance to all key stakeholder groups in Saudi Arabia. The hospitals’ participation in this study was voluntary.

Data from the hospital, including ward- and patient-level data, were collected in accordance with the Global PPS design [37]. The hospital information included the number of patients hospitalized, their gender, age, weight, length of hospitalization, therapy, and comorbidities. In addition, for those patients prescribed antimicrobials, more information was collected. This included the number of antibiotics prescribed, their route, and their ATC classification. Alongside this, the indication including the organ system involved, any surgical procedures undertaken, any catheterization or intubation, or any culture reports requested were included. The prescribed antibiotics were further broken down into ‘Access’, ‘Watch’, and ‘Reserve’ antibiotics, with a suggested target of 60% of prescriptions being ‘Access’ antibiotics to limit AMR [39,47,48]. A web-based program developed by the University of Antwerp was utilized for data entry, validation, and reporting [1].

### 4.2. Inclusion and Exclusion Criteria

This PPS study included patients receiving at least one antimicrobial (antibacterial, antifungal, or antiviral) for systemic use related to their clinical condition. However, patients in short-stay, discharged patients, patients in emergency, outpatient departments, and long-term care units were excluded, similar to other PPS studies [1].

### 4.3. Data Collection

As previously stated, this study utilized a structured data collection tool (Global PPS) to gather information on current antimicrobial prescribing patterns. Patients’ prescribing charts and medical case notes were examined for detailed information on the variables of interest. This study included all patients hospitalized overnight and still in the ward at 08:00 a.m. on the day of the PPS, and all prescribed antimicrobials at the time of the survey. The data were thoroughly reviewed for accuracy and completeness, and definitions for medical treatments, including surgical prophylaxis, healthcare-associated infections (HAI), and community-acquired infections were obtained from the Global PPS methodology handbook. There was no direct interaction with patients at any stage of data collection, in line with other PPS studies. Similarly, there was no contact with any physicians managing these patients, with any data taken directly from the patient’s notes. The survey was conducted in the entire hospital during the study period, but data were collected in one specific ward on the same day. This was done to ensure that all eligible patients were surveyed within the recommended data collection period. Additionally, there were multiple data collector teams to collect data from the hospitals. Therefore, the completion time of one hospital depends on the number of wards in the hospital and the number of data collection teams. While it might not have been possible to synchronize the data collection to occur on precisely the same date in all five hospitals, we strictly adhered to the requirement of completing the survey within the recommended three-week period from the initial data collection day. The data were entered into a web-based Global PPS program.

### 4.4. Statistical Analysis

Data analysis was performed using Microsoft Excel (2019) and the Statistical Package for the Social Sciences (SPSS) software (version 25.0), and descriptive statistics were applied.

### 4.5. Ethical Approval

This study was reviewed and discussed by the IRB Committee and was approved according to ICH GCP guidelines (IRB Number: H-02-K-076-0321-494).

There was no direct contact with patients to obtain their permission to access their notes, with data collected retrospectively from patients’ medical records and notes. Consequently, the approval committee exempted written informed consent from potential participants. This is in line with similar studies undertaken by the coauthors. In addition, no personal patient information was collected, such as their name, address, or telephone number, to maintain confidentiality, with each patient was given a study number. This is in line with previous PPS studies conducted by the coauthors.

## 5. Conclusions

This study suggests high prescription rates for antibiotics among patients hospitalized in Saudi Arabia during the COVID-19 pandemic, especially from the ‘Watch’ group of antibiotics. These findings are in line with previous studies conducted in different countries, and highlight the need for continued efforts to optimize the rational use of antimicrobials in hospitals in Saudi Arabia through implementing ASPs. We will be following this up in future research projects to reduce excessive prescribing of ‘Watch’ antibiotics.

## Figures and Tables

**Table 1:** Overall characteristics of the study population.

Characteristics	*N*	%, Range and Mean
Total beds (number)	930	
Hospitalized patients	897	
Number of treated patients and percentage	518 (57.7%)	
Number of prescribed antibiotics	982	
Number of antibiotics per patient (median)	2	Range: 1–6; Mean: 1.9
Gender		
Male	260	50.2%
Female	258	49.8%
Age (median)	33 years	Range: 1 day–97 years; Mean: 34.3 years
Weight (median)	65 kg	Range: 2–165 kg; Mean: 55.7 kg
Length of Hospitalization (median)	7 days	Range: 1–30 days; Mean: 10.8 days
No. of Antibiotics		
1	244	47.11%
2	164	31.75%
>2	110	21.14%
Comorbidities	164	31.7%
Immunosuppressant use	28	5.4%
Surgical procedures	156	30.1%
Catheterization		
Peripheral	216	41.7%
Central	66	12.7%
Urinary	42	8.1%
Intubation		
Nasogastric tube	62	12.0%
Endotracheal tube	18	3.5%
COVID-19		
Yes	86	16.6%
No	432	83.4%

**Table 2:** Culture reports and sites of infection.

Parameters	*N*	%
Culture Reports	174	36.5
Sample		
Blood	116	22.4
CSF	6	1.2
Sputum	8	1.5
Urine	24	4.6
Wound swab	20	3.9
Resistance Microbes		
Carbapenem-resistant nonfermenter Gram-negative	20	3.9
ESBL-producing nonfermenter Gram-negative bacilli	8	1.6
3rd generation cephalosporin-resistant	22	4.2
Carbapenem-resistant Enterobacteriaceae	10	2.0
Vancomycin-resistant Enterococci (VRE)	6	1.2
Targeted treatment against other MDR organisms	16	3.1
ESBL-producing enterobacteria	12	2.4
Methicillin-resistant Staphylococcus aureus (MRSA)	12	2.4
Ceftazidime/avibactam resistance	2	0.4
Type of Infection		
Community-acquired infection	318	61.4
Hospital-acquired infection	158	30.5
Surgical/medical prophylaxis	42	8.1

CSF: Cerebrospinal fluid, ESBL: Extended spectrum beta lactamase.

**Table 3:** Most commonly prescribed antimicrobials in different indication.

Antibiotics	Total		Community-Acquired Infection		Hospital-Acquired Infection	
	*N*	%	*N*	%	*N*	%
Ceftriaxone (W)	136	13.91	98	16.4	28	8.5
Piperacillin/Tazobactam (W)	122	12.47	78	13.1	40	12.1
Vancomycin (W)	106	10.84	48	8.1	56	17.11
Meropenem (W)	82	8.38	38	6.4	44	13.3
Cefazolin (A)	74	7.57	38	6.4	22	6.7
Ampicillin (A)	74	7.57	32	5.4	36	10.9
Gentamicin (A)	44	4.50	14	2.3	26	7.9
ATT (W)	42	4.29	32	5.4	10	3.1
Metronidazole (A)	36	3.68	30	5.0	6	1.8
Co-amoxiclav (A)	26	2.66	18	3.0	6	1.8
Ceftazidime (W)	26	2.66	18	3.0	6	1.8
Clindamycin (A)	24	2.45	20	3.4	2	0.6
Ciprofloxacin (W)	22	2.25	16	2.7	6	1.8
Azithromycin (W)	20	2.04	14	2.3	6	1.8
Cefuroxime (W)	18	1.84	12	2.0	6	1.8
Cefotaxime (W)	18	1.84	12	2.0	6	1.8
Levofloxacin (W)	16	1.64	14	2.3	2	0.6
Amikacin (A)	14	1.43	6	1.0	8	2.4
Cefepime (W)	14	1.43	8	1.3	2	0.6
Colistin (R)	14	1.43	12	2.0	2	0.6
Miscellaneous	54	5.52	38	6.4	10	3.1
Total	982	100	596	100	330	100

A: ‘Access’; ‘W’: Watch; R: ‘Reserve’—based on the AWaRe classification [47,48]. The % is calculated column-wise.

**Table 4:** Antimicrobials prescribed among COVID and non-COVID patients.

Antibiotics	Total		COVID-19		Non COVID-19	
	*N*	%	*N*	%	*N*	%
Ceftriaxone (W)	136	13.91	20	10.75	116	14.57
Piperacillin/Tazobactam (W)	122	12.47	34	18.28	88	11.06
Vancomycin (W)	106	10.84	20	10.75	86	10.80
Meropenem (W)	82	8.38	20	10.75	62	7.79
Cefazolin (A)	74	7.57	4	2.15	70	8.79
Ampicillin (A)	74	7.57	6	3.23	68	8.54
Gentamicin (A)	44	4.50	2	1.08	42	5.28
ATT (W)	42	4.29	8	4.30	34	4.27
Metronidazole (A)	36	3.68	6	3.23	30	3.77
Co-amoxiclav (A)	26	2.66	2	1.08	24	3.02
Ceftazidime (W)	26	2.66	4	2.15	22	2.76
Clindamycin (A)	24	2.45	6	3.23	18	2.26
Ciprofloxacin (W)	22	2.25	0	0.00	22	2.76
Azithromycin (W)	20	2.04	16	8.60	4	0.50
Cefuroxime (W)	18	1.84	0	0.00	18	2.26
Cefotaxime (W)	18	1.84	4	2.15	14	1.76
Levofloxacin (W)	16	1.64	8	4.30	8	1.01
Amikacin (A)	14	1.43	2	1.08	12	1.51
Cefepime (W)	14	1.43	4	2.15	10	1.26
Colistin (R)	14	1.43	2	1.08	12	1.51
Miscellaneous	54	5.52	18	9.68	36	4.52
Total	982	100	186	100	796	100

A: ‘Access’; ‘W’: Watch; R: ‘Reserve’—based on the AWaRe classification [47,48]. The % is calculated column wise.

**Table 5:** Most commonly prescribed antimicrobials according to organ system.

Antibiotics	Blood	RTI	SSTI	GIT	UTI	CNS	CVS	OBGY
Ceftriaxone (W)	16	44	10	26	12	20	6	0
Piperacillin/Tazobactam (W)	38	24	24	14	10	4	2	2
Vancomycin (W)	38	14	10	6	10	12	4	2
Meropenem (W)	30	14	6	4	12	4	2	4
Cefazolin (A)	8	6	24	10	8	2	2	6
Ampicillin (A)	40	18	6	6	0	2	0	2
Gentamicin (A)	28	6	4	0	2	2	0	2
ATT (W)	0	42	0	0	0	0	0	0
Metronidazole (A)	6	2	8	18	2	0	0	0
Co-amoxiclav (A)	4	0	14	4	2	0	0	0
Ceftazidime (W)	14	2	4	0	4	2	0	0
Clindamycin (A)	2	4	8	2	2	6	0	0
Ciprofloxacin (W)	8	0	4	0	2	4	2	2
Azithromycin (W)	4	12	2	0	0	0	0	2
Cefuroxime (W)	6	2	2	8	0	0	0	0
Cefotaxime (W)	6	6	4	0	0	2	0	0
Levofloxacin (W)	4	10	0	0	0	2	0	0
Amikacin (A)	6	0	0	2	2	0	0	0
Cefepime (W)	0	8	4	0	0	0	0	2
Colistin (R)	2	0	6	0	2	0	0	2
Total	260	214	140	100	70	62	18	26

CNS: Central nervous system; CVS: cardiovascular system; GIT: gastrointestinal tract; OBGY: obstetric or gynecological; SSTI: skin and soft tissues infection; RTI: respiratory tract infections, UTI: urinary tract infections. A: ‘Access’; ‘W’: Watch; R: ‘Reserve’—based on the AWaRe classification [47,48].

**Table 6:** Top three most commonly prescribed antimicrobials according to organ system.

Organ System	*N*	Antibiotic 1	*N*	Antibiotic 2	*N*	Antibiotic 3	*N*
Blood	120	Ampicillin (A)	40	Piperacillin/Tazobactam (W)	38	Vancomycin (W)	38
RTI	114	Ceftriaxone (W)	44	ATT (W)	42	Piperacillin/Tazobactam (W)	24
SSTI	86	Cefazolin (A)	24	Piperacillin/Tazobactam (W)	24	Co-amoxiclav (A)	14
GIT	64	Ceftriaxone (W)	26	Metronidazole (A)	18	Piperacillin/Tazobactam (W)	14
UTI	40	Meropenem (W)	12	Ceftriaxone (W)	12	Piperacillin/Tazobactam (W)	10
CNS	38	Ceftriaxone (W)	20	Vancomycin (W)	12	Clindamycin (A)	8
OBGY	18	Cefazolin (A)	6	Meropenem (W)	4	Piperacillin/Tazobactam (W)	2
CVS	14	Ceftriaxone (W)	6	Vancomycin (W)	4	Cefazolin (A)	2
Others	24	Vancomycin(W)	10	Cefazolin (A)	8	Meropenem (W)	6
Total	518		188		162		118

CNS: Central nervous system; CVS: cardiovascular system; GIT: gastrointestinal tract; OBGY: obstetric or gynecological; SSTI: skin and soft tissues infection; RTI: respiratory tract infections, UTI: urinary tract infections. A: ‘Access’; ‘W’: Watch; R: ‘Reserve’—based on the AWaRe classification [47,48].

## Data Availability

Data are contained within the article.
